# Molecular Pathogenesis and New Therapeutic Dimensions for Spinal Muscular Atrophy

**DOI:** 10.3390/biology11060894

**Published:** 2022-06-10

**Authors:** Andrés López-Cortés, Gabriela Echeverría-Garcés, María José Ramos-Medina

**Affiliations:** 1 Programa de Investigación en Salud Global, Facultad de Ciencias de la Salud, Universidad Internacional SEK, Quito 170302, Ecuador; 2 Facultad de Medicina, Universidad de Las Américas, Quito 170124, Ecuador; 3 Latin American Network for the Implementation and Validation of Clinical Pharmacogenomics Guidelines (RELIVAF-CYTED), 28001 Madrid, Spain; mgecheverriag@gmail.com (G.E.-G.); mary.pepita967@gmail.com (M.J.R.-M.)

**Keywords:** spinal muscular atrophy, *SMN1*, *SMN2*, recessive genetic disease

## Abstract

**Simple Summary:**

Globally, 5q spinal muscular atrophy (SMA) is one of the most common pediatric autosomal recessive neuromuscular diseases, with a prevalence of ~1–2 per 100,000 inhabitants and an incidence of ~1 per 10,000 live births, which makes it the most common cause of infant genetic mortality. It is characterized primarily by the progressive degeneration of α-motor neurons in the ventral grey horn of the spinal cord. Over the years, the development of innovative 5q SMN replacement and/or splice modulation strategies has provided therapeutic options for children diagnosed with 5q SMA, delaying the progression of 5q SMA and increasing patient survival, despite its extremely high cost. In this review, we provide an overview of the rapidly evolving therapeutic landscape for SMA, including SMN-targeted therapies, SMN-independent therapies, and combinational therapies that are likely to be key for the development of treatments that are effective across a patient’s lifespan.

**Abstract:**

The condition known as 5q spinal muscular atrophy (SMA) is a devastating autosomal recessive neuromuscular disease caused by a deficiency of the ubiquitous protein survival of motor neuron (SMN), which is encoded by the *SMN1* and *SMN2* genes. It is one of the most common pediatric recessive genetic diseases, and it represents the most common cause of hereditary infant mortality. After decades of intensive basic and clinical research efforts, and improvements in the standard of care, successful therapeutic milestones have been developed, delaying the progression of 5q SMA and increasing patient survival. At the same time, promising data from early-stage clinical trials have indicated that additional therapeutic options are likely to emerge in the near future. Here, we provide updated information on the molecular underpinnings of SMA; we also provide an overview of the rapidly evolving therapeutic landscape for SMA, including SMN-targeted therapies, SMN-independent therapies, and combinational therapies that are likely to be key for the development of treatments that are effective across a patient’s lifespan.

## 1. Introduction

The condition known as 5q spinal muscular atrophy (SMA) is an autosomal recessive neuromuscular disease characterized primarily by the progressive degeneration of α-motor neurons in the ventral grey horn of the spinal cord [1,2]. It is one of the most common pediatric recessive genetic diseases, with a prevalence of ~1–2 per 100,000 inhabitants and an incidence of ~1 per 10,000 live births, which makes it the most common cause of infant genetic mortality [3]. Additionally, Dangouloff et al. have identified nine SMA newborn screening (NBS) programs worldwide that have so far detected 288 neonates with SMA out of 3,674,277 newborns screened (incidence rate of 1:12,758) [4]. The epidemiology of these 9 NBSs is fully detailed in Table 1.

The cause of 5q SMA is a deficiency of the ubiquitous protein survival of motor neuron (SMN), which is encoded by the SMN genes, *SMN1* and *SMN2* [12]. This molecular deficiency leads to muscle wasting and weakness, feeding and respiratory difficulties, paralysis, and, in the most severe types, death [13]. Several 5q SMA types have been described; SMA-I is responsible for ~60% of cases and is the most severe, usually leading to death before the age of 2 years [1]. The other 5q SMA variants include types II, III, IV, and 0.

Over the years, SMA therapies have been supportive and have had no effect on the underlying disease process. However, intensive basic and clinical research efforts and improvements in standard of care have developed successful therapeutic milestones, slowing SMA progression and increasing patient survival [14,15]. In this review, we update the information on the molecular pathogenesis of SMA and discuss the challenges that are arising as a result of the novel therapeutic strategies.

## 2. Variability of Clinical Features, Phenotypes, and Severity

Spinal muscular atrophy is divided into five clinical types based on age of onset and severity [1,16]. First, 5q SMA-I, also known as Werding–Hoffman disease (Online Mendelian Inheritance in Man [OMIM] number 253300), is the most severe and common type (~60% of patients diagnosed), which is distinguished by profound weakness within the first 6 months of life and death within the first 2 years of life [17]. These patients present hypotonia, inability to sit without support, symmetrical flaccid paralysis, no control of head movement, and fatal respiratory failure [1,18]. Second, 5q SMA-II, also known as Dubowitz disease (OMIM number 253550), is of intermediate severity and characterized by the onset of proximal muscle weakness before 18 months of age. These patients can sit unsupported, but none can walk independently. Respiratory complications are a frequent cause of death during adolescence [18]. Third, 5q SMA-III, also known as Kugelberg–Welander disease (OMIM number 253400), is distinguished by profound symptom heterogeneity. The onset of disease in these patients most often occurs between the ages of 18 months and 18 years, meaning they achieve the ability to walk unassisted. However, patients with an age of onset before 3 years lose the ability to walk in childhood [1]. Fourth, 5q SMA-IV, or so-called adult-type, affects <1% of cases in which onset is after the age of 18 years [19]. This mildest form of SMA brings to patients a normal life expectancy and modest disability. Fifth, 5q SMA type 0 is an extremely rare variant with onset in utero and poor survival [1]. Lastly, non-SMN-related SMA types include spinal and bulbar muscular atrophy (Kennedy’s disease), X-linked disease, distal SMA, and SMA with respiratory distress [18].

## 3. Clinical History and Management

A proactive disease management approach requires early implementation of standard of care therapies in SMA patients, including respiratory, gastrointestinal, nutritional, and orthopedic support. Pulmonary-related complications such as deficient clearance of airway secretions, chest infections, hypoventilation, and respiratory failure are the main contributing factors to disease morbidity and mortality, mainly in 5q SMA type I and type II patients [20]. Support in airway secretion clearance can be provided by manual chest physiotherapy, mechanical insufflation–exsufflation therapy, and oral suctioning [21]. Shortness of breath detected during a sleep study or from gas-exchange assessments can help determine if non-invasive ventilation (NIV) is required before respiratory distress presents and should be performed by a trained professional [21]. These patients usually have a weak cough that results in mucus accumulation and plugging, increasing the risk of aspiration, low oxygen blood levels, and chest infections, which can be monitored with an oximeter and airway clearance supportive methods [20]. If disease progresses and NIV becomes insufficient, patients can decide to undergo tracheotomy. Other palliative and preventive measures include viral and influenza immunizations [21].

Metabolic, endocrine, and gastrointestinal disorders are also common in the SMA population, requiring a professional adjustment of calorie, liquid, and macro- and micronutrient needs. Palliative care to manage complications of the gastrointestinal tract includes adjusting food consistency to a semi-solid and thickened liquid diet to lessen swallowing difficulties, reduce risk of aspiration-related chest infections, and improve digestion [22], together with anti-reflux and bowel regulation medications [23]. Malnutrition can originate due to debilitation of masticatory muscles and dysphagia, increasing muscle mass loss. Therefore, gastrostomy tube placement and Nissen fundoplication are recommended surgical interventions [23] to improve nutrition and quality of life and reduce mortality risk [24]. Micronutrient deficiencies also result from decreased energy intake. Subsequently, vitamin D- and calcium-supplemented diets can be beneficial, since they increase mineral bone density [23], which is associated with a reduced risk of fractures, osteoporosis, and scoliosis [25]. Vitamin B12 and folate supplementation have also been recommended due to their role in SMN methylation [26].

Muscle weakness and impaired mobility are characteristic features of SMA, resulting in subsequent musculoskeletal alterations. Regular stretching and physical therapy are provided using orthoses and splints, cervical and thoracic braces to stabilize posture, and other positioning supportive methods in order to optimize function and prevent contractures and other common mobility-related complications [23]. Wheelchair support can be initiated in ambulatory SMA patients as early as 18 to 24 months, or standing frames can be used by patients who are still able to maintain some weight on their legs [22]. Physical activities such as swimming and aerobic and resistance exercises are recommended among patients with milder phenotypes to maintain and improve mobility functions and endurance [23]. Lastly, scoliosis can develop in patients who are able to sit and walk. Spinal orthoses are the primary management treatment, although some patients may require surgical intervention and bracing to prevent future cage deformity and respiratory obstruction caused by spinal deformities, in which case it is not recommended before 4 years of age [23].

## 4. Molecular Genetics and Pathogenesis

**Figure 1:**
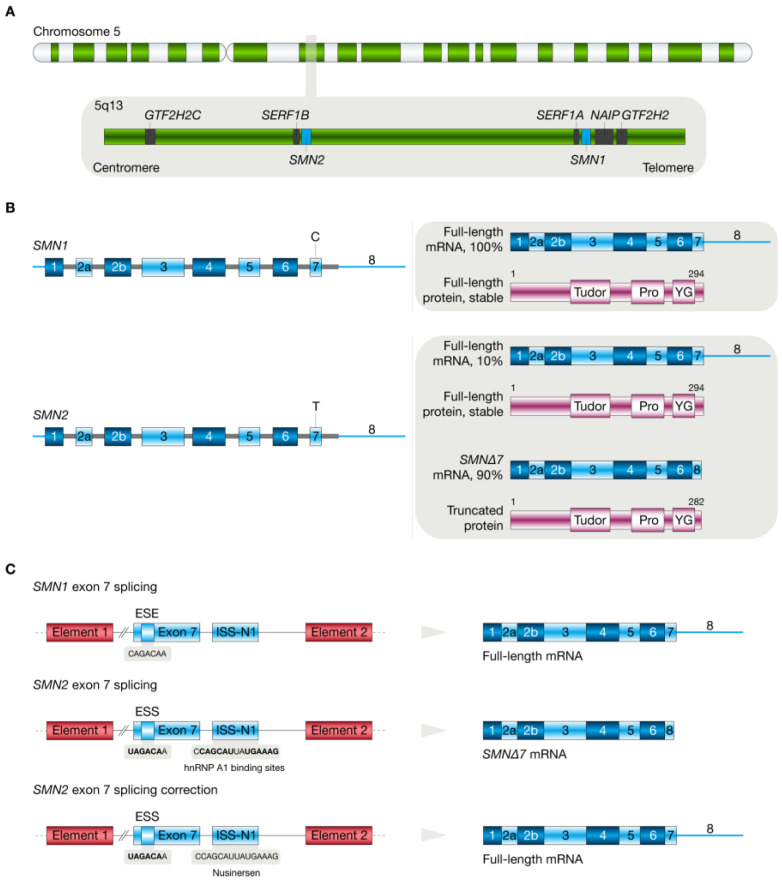
Genetic overview of spinal muscular atrophy. (**A**) Human chromosome 5q13 is encompassed by a telomeric *SMN1* gene and a centromeric *SMN2* gene. (**B**) Splicing of *SMN1* pre-mRNA produces full-length mRNA that is translated into functional SMN protein containing a Tudor domain, a proline-rich region, and a YG-box domain. On the other hand, splicing of *SMN2* pre-mRNA translates into a functional SMN protein in ~10% of cases, and splicing of *SMN2* pre-mRNA produces *SMN2* mRNA without exon 7 (SMN2Δ7), translating a truncated protein easily degraded in ~90% of cases. Lastly, (**C**) shows exon 7 splicing in *SMN1* and *SMN2* genes. The exonic splice enhancer (ESE) promotes the translation of full-length SMN1 protein. The C-to-T transition leads to the formation of an exonic splice suppressor (ESS), which binds the heterogeneous nuclear ribonucleoprotein A1 (hnRNP A1) and translates an unstable SMNΔ7 protein. Antisense oligonucleotides (i.e., nusinersen) target the intronic splice silencer (ISS-N1), preventing the binding of hnRNP A1 and promoting exon 7 inclusion and subsequent translation of full-length SMN protein.

The *SMN* gene is part of a 500 kilobases (kb) inverted duplication on chromosome 5 at locus 5q13 [27]. In 1995, *SMN* was identified as the SMA disease-causing gene, which is present in multiple copies in the human genome: one copy of *SMN1* is located in the telomeric region, and several copies of *SMN2* are located in the centromeric region [27]. *SMN1* and *SMN2* are encompassed by nine exons (1, 2a, 2b, 3, 4, 5, 6, 7, and 8) and eight introns that span a genomic region of about 20 kb. The two genes vary by eight nucleotides from each other, five of which are intronic, and three of which occur in the last three exons [18]. More than 95% of 5q SMA patients have a homozygous disruption of *SMN1* by deletion, mutation, or rearrangement, which leads to loss of SMN expression [28,29]. However, the 5q SMA severity is determined by the variable copy number of *SMN2*, which is paralogous to *SMN1*. *SMN2* undergoes alternative splicing and produces truncated mRNA isoform in which exon seven is absent. A translationally silent cytosine (C)-to-thymine (T) nucleotide transition (c.840 C > T) at position six of exon seven is responsible for the alternatively spliced isoform causing removal of exon seven in *SMN2*, resulting in shortened mRNA that encodes an unstable and non-functional SMNΔ7 protein (282 amino acids, 30.5 kDa) that is rapidly degraded by the proteasome pathway [30,31,32]. *SMN2* exon seven skipping occurs because the C to T transition impedes an exonic splicing enhancer (ESE) site where SF2/ASF binds [33] and produces a novel exonic splicing silencer (ESS) site where the splicing factors heterogeneous nuclear ribonucleoprotein (hnRNP) A1/A2 bind [34]. An estimated 10% of *SMN2* precursor messenger RNA (pre-mRNA) is correctly spliced and translated into full-length SMN protein (294 amino acids, 32 kDa) [35,36]. Nevertheless, the low level of SMN protein is not enough to sustain the survival of motor neurons of the spinal cord [18] (Figure 1).

### 4.1. Predominance of Pathogenic Variants in the SMN Genes

**Figure 2:**
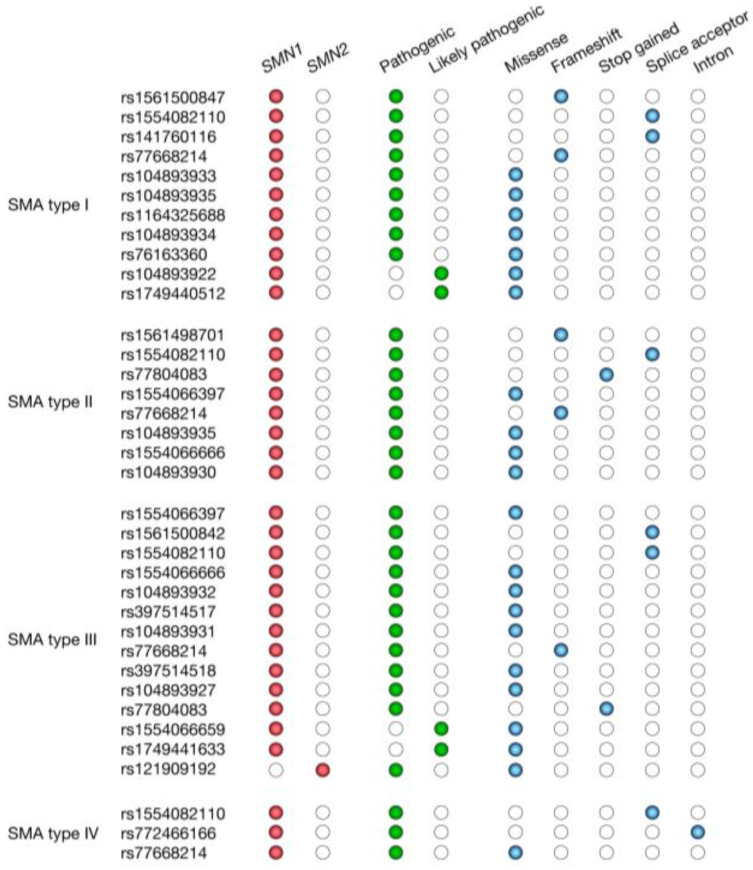
Less frequently reported genetic variants involved in spinal muscular atrophy. The figure shows pathogenic and likely pathogenic variants reported in the *SMN1* and *SMN2* genes with their respective consequence type (missense, frameshift, stop gained, splice acceptor, and intron variant).

The majority of 5q SMA cases are caused by an absence of the *SMN1* gene. Ninety-five percent of 5q-SMA-affected individuals have a homozygous deletion of *SMN1* exon 7 or gene conversion from *SMN1* to *SMN2*, and most of the remaining 5% are compound heterozygotes for an *SMN1* exon 7 deletion and an *SMN1* point mutation. However, other pathogenic and likely pathogenic variants reported in the *SMN1* and *SMN2* genes are involved in the 5q SMA types I, II, III, and IV. Subsequently, our integrated analysis using VarSome (https://varsome.com/, accessed on 8 February 2022), a search engine for human genomic variation [37] where relevant data from the UniProt (https://www.uniprot.org/, accessed on 8 February 2022) [38] and the ClinVar (https://www.ncbi.nlm.nih.gov/clinvar/, accessed on 8 February 2022) [39] databases are collected, and the Open Targets Platform (https://www.targetvalidation.org, accessed on 15 February 2022), a comprehensive and robust data integrator that estimates the pathogenic association between genetic variants and SMA [40], reveals that *SMN1* carries 21 pathogenic variants (13 missense, 3 frameshift, 3 splice acceptor, 1 stop gained, and 1 intron variants) and 4 likely pathogenic variants (missense variants) associated with 5q SMA. Additionally, *SMN2* carries 1 pathogenic variant (missense variant) associated with 5q SMA (Figure 2).

### 4.2. The SMN Complex

SMN is an RNA-binding protein that performs multiple essential cellular functions and processes, most notably playing a critical role in the small nuclear ribonucleoprotein (snRNP) complex assembly in the cytoplasm [41]. The large SMN complex (40S to 80S in sucrose gradient sedimentation) is involved in the assembly, metabolism, and transport of diverse ribonucleoproteins (i.e., snRNPs, telomerase RNPs, and microRNPs) [42] and is encompassed by the SMN protein and seven additional proteins (Gemin2–8) [43,44,45,46,47,48,49]. The Gemins bind to and co-localize with SMN in the cytoplasm, nucleus, and nuclear gems (Gemini of Cajal bodies) [50]. Gemin2, -3, -5, and -7 interact directly with SMN, while Gemin4 and -6 may be indirectly associated, as they require binding to Gemin3 and -7, respectively [51]. Notably, the SMN complex is a molecular chaperone whose phosphorylation regulates the biogenesis and function of snRNPs that are involved in splicing [50,52].

### 4.3. Spliceosomal U Small Nuclear Ribonucleoprotein Assembly

**Figure 3:**
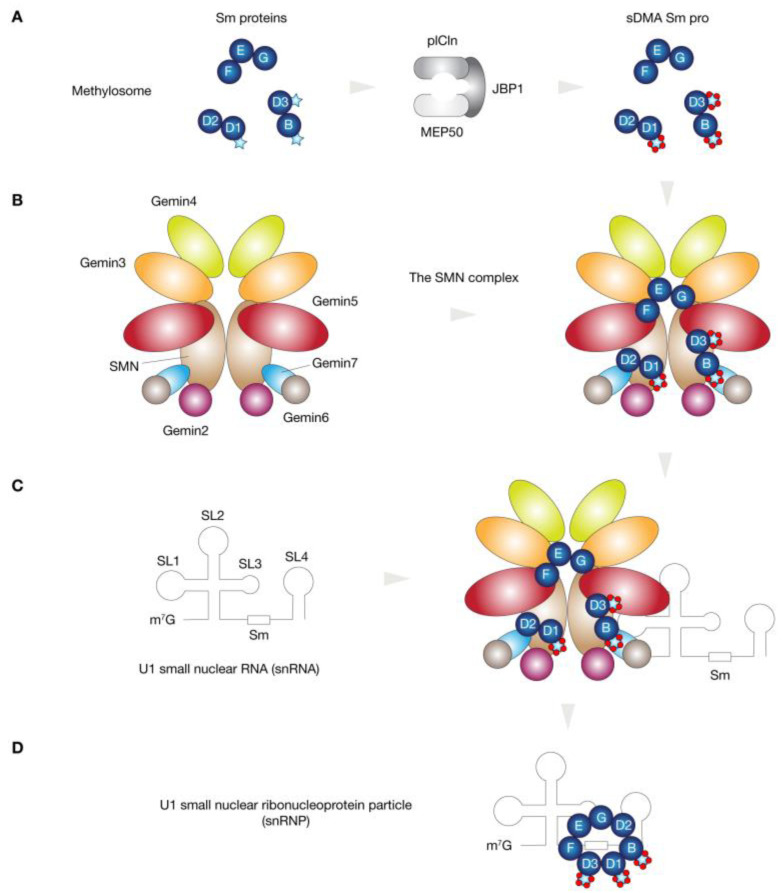
A schematic representation of U1 small nuclear ribonucleoprotein particle (snRNP) assembly mediated by the SMN complex. (**A**) The methylosome modifies specific arginines of several Sm proteins to symmetric di-methylarginines (sDMA) Sm proteins. (**B**) In the SMN complex, Gemins 2, 3, 5 and 7 bind directly to SMN, while Gemins 4 and 6 have direct interactions with Gemins 3 and 7, respectively. (**C**) Sm proteins associate with the SMN complex, allowing the direct binding of the SMN complex to specific domains of U1 small nuclear RNA (snRNA). (**D**) The SMN complex with bound Sm proteins is the active form for snRNP assembly that is crucial for pre-mRNA processing to mRNAs.

The pre-mRNA splicing is a key process for the effective execution of gene expression and is catalyzed by the spliceosome, a multi-megadalton RNA–protein complex comprising five snRNPs and more than one hundred proteins [53]. There are two types of spliceosomes that coexist and are essential in higher eukaryotes; the major spliceosomes (U1, U2, U4, U5, and U6 snRNAs) remove >99.5% of introns (U2-type), while the minor spliceosomes (U11, U12, U4atac, U5, and U6atac snRNAs) remove the rest of introns (U12-type) [54,55]. The consensus sequences of the 5′ splice site (SS), the branch site (BS), and the 3′ SS are more conserved in U12-type introns than in U2-type introns [54,56]. Regarding snRNP biogenesis, it begins with the transcription of U snRNAs in the nucleus and their subsequent export to the cytoplasm. The SMN protein facilitates the assembly of Sm proteins (B/B’, D1, D2, D3, E, F, and G) and Sm-like (Lsm) proteins (Lsm2–8) into a stable heptameric ring (the Sm core) on a uridine-rich sequence motif (the Sm site) of the snRNA to produce functional snRNPs, also known as spliceosomes [57,58,59,60] (Figure 3).

## 5. Therapeutic Advances in SMA

**Figure 4:**
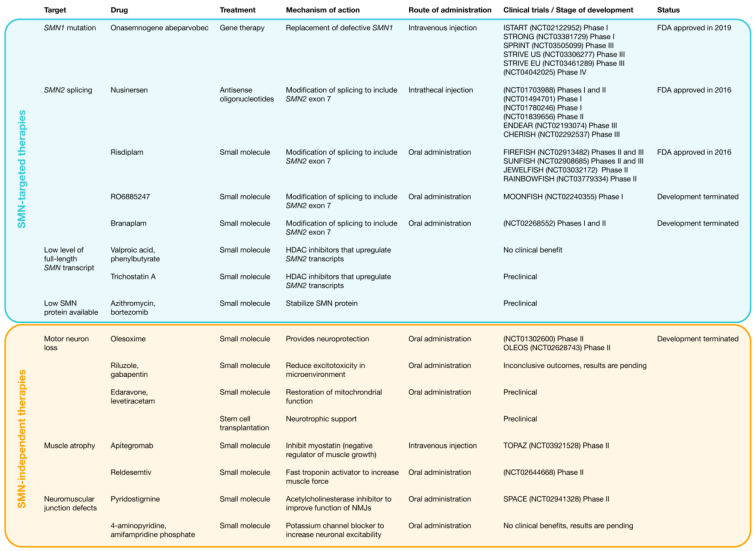
Landscape of targets, drugs, therapeutic strategies, and treatments for SMA. Therapeutic strategies have focused on SMN-dependent approaches, such as the modification of *SMN2* splicing through antisense oligonucleotides and small molecules, the increment of *SMN* transcripts through histone deacetylase (HDAC) inhibitors, and replacement of defective *SMN1* through gene therapy, and SMN-independent approaches, such as neuroprotection, cell therapy through stem cell transplantation, muscle enhancement through myostatin inhibitors and fast troponin activators, and the improved function of neuromuscular junction.

Despite major advance in treatment options for 5q SMA, this monogenic disorder remains an incurable disease. Therapeutic strategies have focused on approaches that increase SMN protein expression, such as the modification of *SMN2* splicing, the increase of *SMN* transcripts, and the replacement of defective *SMN1*, and approaches that are independent of SMN protein, such as neuroprotection, stem cell therapy, muscle enhancement, and the improved function of neuromuscular junction [16] (Figure 4).

### 5.1. Modification of SMN2 Splicing to Include Exon 7

Identification of a genetic deletion in the *SMN1* gene in the majority of 5q SMA patients and its resulting decrease in SMN protein [27] provided a rationale for aiming to restore SMN protein levels by manipulating the *SMN2* paralogue. Given that exon 7 exclusion from the pre-mRNA *SMN2* transcript results in a truncated version of the SMN protein that is degraded [32], numerous studies elucidated several *SMN2*-splicing regulation sites within exon 7 and its neighbor introns that have been assessed as potential therapeutic targets to promote exon 7 inclusion [61].

#### 5.1.1. Antisense Oligonucleotides

Antisense oligonucleotides (ASOs) are single-stranded nucleic acids that can alter pre-mRNA splicing by complementary binding to splicing regulatory elements such as enhancer and silencer target sequences at exonic and intronic regions. Among these, targeting intronic splicing silencer (ISS)-N1, a 15-nucleotide inhibitory sequence downstream of the 5′ end of the *SMN2* intron 7, promotes *SMN2* pre-mRNA exon 7 inclusion [62] with a higher efficacy than targeting other splicing elements [61]. Proof of concept has been demonstrated by a significant increase in full-length *SMN2* pre-mRNA and higher levels of SMN protein in motor neurons, observed after antisense-mediated targeting of ISS-N1 in transgenic mouse models of SMA [61,63,64] and in human clinical trials [65]. Nusinersen (Spinraza; Biogen, Cambridge, MA, USA), an ASO that binds to the ISS-N1 repressive element and inhibits the binding of hnRNP A1 (a splicing repressive element) to allow *SMN2* pre-mRNA exon 7 inclusion, is the first ASO-based therapy for the treatment of 5q SMA that received approval by the US Food and Drug Administration (FDA) agency in 2016 [66] and in Europe by the European Medicines Agency (EMA) in 2017 [67]. Encouraging results from phase III clinical trials in 5q SMA type I infants and later-onset 5q SMA type II and type III patients, known as the ENDEAR and CHERISH studies, respectively, accelerated the approval of nusinersen [68,69]. A total of 51% of nusinersen-treated patients with infantile-onset 5q SMA achieved a motor milestone, showed improvements in neuromuscular functions, and had a higher likelihood of survival compared to the control group [68]. Improvements in motor functions were also confirmed in treated patients with later-onset 5q SMA, in contrast to a deterioration of motor skills observed in untreated patients [69]. Achieved motor milestones are maintained over time, and nusinersen has proved to be beneficial across patients with a wide range of 5q SMA phenotypes [70]. Moreover, nusinersen is well-tolerated and has a satisfactory safety profile, with headache and backache being the most common side effects reported after treatment and respiratory events as the most severe adverse events (AEs), although this is considered to be related to disease progression rather than due to treatment [65,71]. However, the best improvements in the clinical course of SMA are observed when nusinersen is administered at an earlier stage of the disease [68,69]. This is currently being investigated by the NURTURE study, a phase II ongoing clinical trial on presymptomatic SMA infants of up to six weeks of age. After 2.9 years of treatment, all patients were alive, and none required permanent ventilation [72]. At the time of this interim analysis, all patients were able to sit independently; nearly 90% of those expected to develop 5q SMA-I and all of those with expected milder phenotypes were able to walk alone; and at least 67% of all patients, irrespective of expected 5q SMA severity, who had two copies of *SMN2* and all patients with three *SMN2* copies achieved a full score in the Children’s Hospital of Philadelphia Infant Test of Neuromuscular Disorders (CHOP.INTEND) motor function test [72]. In contrast, delaying treatment up to 7 months of age seems to decrease patient survival by 25%, and no full score improvement of motor functions is reported 3 years post-treatment [73]. The long-term benefits and limitations of administering nusinersen at a later symptom onset remain to be revealed by results from the SHINE clinical trial, which includes patients from former trials, including the ENDEAR and CHERISH studies. Nusinersen is at an advanced stage compared to other available therapies, with over 8000 SMA patients treated globally [74]. Nevertheless, its limitations include the inability of ASOs to cross the blood–brain barrier (BBB) and a required continued administration into the spinal cord via intrathecal injections. Although nusinersen effects on the central nervous system (CNS) are encouraging, in vivo studies have shown best outcomes when the ASO is distributed both in the CNS and in peripheral organs [75]. Moreover, several doses are required during the initial part of treatment, making this therapy invasive and challenging due to its delivery method. The development of a peptide-conjugated ASO, which is distributed systemically and in the CNS, has shown enhanced results in transgenic mice compared to standard ASOs and may offer an alternative mode of administration [76].

#### 5.1.2. Small Molecules

Another strategy to modify *SMN2* splicing is through the use of small molecules. An in vitro screening study revealed small molecule splicing modifiers, RG7800 and RG7916, with high specificity for *SMN2* pre-mRNA [77] that promote exon 7 inclusion by interacting with *SMN2* transcript splicing elements and by stabilizing binding of the U1 snRNP spliceosome component at the 5′ splice site of exon 7 [78,79]. These are orally bioavailable, and an increase in *SMN* levels both in the CNS and periphery of animal models treated with these compounds [77,78,80] demonstrates that small molecules can circumvent the major challenges of ASO-based therapy. RG7800 development was terminated before completion of a phase I clinical trial due to toxicity-related events reported in an animal study [81]. Another RNA splicing regulator of *SMN2* is LMI070 (Branaplam), the development of which has recently been discontinued after a single phase I/II clinical trial due to the rapid advances in other 5q SMA therapies rather than due to safety concerns [82]. On the other hand, promising results from clinical trials for RG7916, known as risdiplam (Evrysdi; Roche, Basel, Switzerland), resulted in its approval by the FDA in 2020 [83]. Risdiplam is currently being investigated in phase II/III clinical trials in patients with infantile-onset 5q SMA (FIREFISH) [84], later-onset 5q SMA (SUNFISH) [85], and patients from a broad 5q SMA phenotype who were previously enrolled in other studies and were treated with AVXS-101, nusinersen, the splicing modifier RO6885247, or olesoxime (JEWELFISH) [86]. Preliminary results show that oral administration of risdiplam is safe and results in increased SMN protein levels, increased likelihood of survival, and improvement of motor skills in 5q SMA patients from a broad range of ages and 5q SMA phenotypes [87,88]. At 2 years follow-up, 83% of treated 5q SMA-I infants were free from permanent ventilation and alive. Moreover, the proportion of treated infants that were able to sit unsupported increased by 50% compared to the first 12 months of treatment, further demonstrating improvement of motor functions over time [88]. Risdiplam is also expected to be tested in presymptomatic 5q SMA infants in a phase II clinical trial currently recruiting participants (RAINBOWFISH) [89].

### 5.2. Replacement of Defective SMN1 through Gene Therapy

Due to the monogenic nature of 5q SMA, another treatment strategy is to restore SMN protein levels by replacing the faulty *SMN1* gene with a functional copy. The relatively small *SMN1* cDNA is placed under the control of a hybrid CMV enhancer/chicken-β-actin promoter, packed into the self-complementary adeno-associated viral vector serotype 9 (scAAV9), and delivered intrathecally or systemically. These features allow an efficient transduction of 60% of the motor neurons [90], without integrating into the host genome, and a fast and continued expression of SMN [91,92]. Favorable results have been observed from preclinical studies in mice, pigs, and non-human primates, demonstrating rescue of phenotype, improvements in motor functions, and increase in lifespan after gene therapy treatment [93,94,95,96]. A first-in-human clinical trial of gene therapy AVXS-101 or onasemnogene abeparvovec (Zolgensma; Novartis, Basel, Switzerland), known as START, was conducted in 15 5q SMA-I infants of up to 6 months of age, who received a single intravenous dose of AVXS-101 and were matched with a historical cohort for comparison. After 3 years of treatment, all patients were alive, and none required permanent ventilation, compared to a mean survival rate of 8% and permanent ventilation before 11 months of age reported in the historical cohort database [91]. Moreover, a rapid improvement in motor functions was observed only 1 month after treatment, and meaningful motor milestones in the sitting, speaking, and feeding categories were achieved [91]. Nevertheless, the best outcomes in motor functions were achieved by those children who received AVXS-101 at an earlier age, presumably before motor neuron deterioration occurred [92]. A multicenter, single-arm phase III trial (STR1VE) of AVXS-101 in infants younger than 6 months also reported encouraging results, as demonstrated by 90% of patients being free from permanent ventilation at 14 months of age and 60% of children being able to sit unsupported at 18 months, both drastically different from the clinical course of the untreated historical group [97]. Onasemnogene abeparvovec, now commercialized under the name of Zolgensma, became the first gene-based therapy for the treatment of SMA that received approval by the FDA in 2019 [98], with more than 1000 patients treated worldwide [99]. Treatment regimen includes a single intravenous dose of 1 × 10^14^ vector genomes/Kg for 30–60 min. The effects of Zolgensma in patients with a broad range of SMA phenotypes are expected to be investigated in a long-term follow-up phase IV clinical trial (NCT04042025) currently in the recruitment stage.

One of the biggest advantages of Zolgensma over other existing 5q SMA treatments is that the treatment regimen requires a single dose easily administered to patients by intravenous injection. On the other hand, 5q SMA patients require several loading doses followed by maintenance doses, which need to be injected in the cerebrospinal fluid if nusinersen is the treatment of choice, or receive daily oral administration of risdiplam throughout life. Secondly, the viral vector used in the gene-based approach can cross the BBB with high efficiency [90] and ensure both CNS and peripheral transduction. Researchers have defined 5q SMA as a multisystemic disease affecting other tissues including but not limited to the heart, pancreas, skeletal muscle, and reproductive system [100,101], highlighting the importance of also increasing SMN levels in non-neuronal tissue. In fact, assessment of the biodistribution of onasemnogene abeparvovec in post-mortem tissue of 5q SMA type I patients has revealed the widespread transduction of tissues after systemic delivery, with the liver, pancreas, intercostal muscle, psoas, and diaphragm being the tissues the highest transduction rates [102], therefore supporting the efficacy of gene therapy for SMA treatment.

Although Zolgensma is considered a well-tolerated treatment among patients, a commonly reported adverse event from clinical trials is liver toxicity, which has been successfully managed with prednisolone treatment [91,97]. Nevertheless, it is worth considering the potential risks associated with high-dose AVV9-SMN delivery. Severe side effects including hepatic toxicity, thrombocytopenia, and systemic shock in non-human primates have been documented after systemic delivery of a similar dose used in human clinical trials of vector-SMN therapy [103]. Moreover, widespread lesions within the peripheral and central nervous systems, particularly degeneration of the dorsal root ganglia (DRG) sensory neurons, have been observed both in non-human primates and piglets [103]. Other concerns include pre-existing anti-AVV9 antibodies in patients, which, despite having been found at relatively low rates in young people [104], have previously led to patient exclusion from therapy [91]. Increasing the number of patients in future clinical trials might reveal the extent of this limitation. Finally, the effects of long-term expression of SMN mediated by the AVV9 vector are still uncertain. Recently, studies in mouse models have reported that overexpression of SMN beyond physiological levels results in dose-pendent neurotoxicity, neurodegeneration, and the formation of SMN cytoplasmic aggregates that sequester snRNPs components, leading to splicing and transcriptomic alterations [105].

Other therapeutic strategies for SMA include the development of *SMN*-independent treatments that aim to ameliorate motor neuron loss and muscle atrophy, the most affected and prominent features of SMA.

### 5.3. Neuroprotection

One of the neuroprotective clinical strategies explored so far aims to prevent motor neuron loss by using small molecules that target different cellular components or pathways to promote motor neuron survival. Among the earliest attempts, repurposing of the drugs Gabapentin and Riluzole for SMA was investigated for their neuroprotective effects by reducing excitotoxicity in the microenvironment. Despite initial encouraging results in a mouse model [106], first-in-human clinical trials of Riluzole failed to show a clinical benefit for SMA [107]. Moreover, a phase II/III clinical trial (NCT00774423) of Riluzole has been conducted in children and adult SMA patients, but results have not been reported. Similarly, Gabapentin clinical trials have shown poor or inconclusive outcomes [108,109]. Olesoxime is a neuroprotective cholesterol-like small molecule that can delay motor neuron death by decreasing the permeability of the outer mitochondrial membrane, inhibiting the release of pro-apoptotic factors [110,111]. A multicenter phase 2 trial in 5q SMA type 2 and type 3 patients found that there were no risks associated with the daily oral administration of olesoxime and that it might help maintain motor functions over time [112]. However, the extension trial (OLEOS) reported a decline in motor function after 52 weeks of treatment [113], resulting in the termination of olesoxime development for SMA, as announced by Roche in 2018. Recently, Edaravone, a drug used in amyotrophic lateral sclerosis, and Levetiracetam, an anti-epileptic, have shown potential clinical benefit in the development of motor neurons [114], restoration of mitochondrial function, and motor neuron survival [115]. However, this has only been tested in an in vitro setting.

### 5.4. Stem Cell Therapy

Stem cell transplantation constitutes another approach to prevent the progressive loss of motor neurons. Stem cells possibly confer neuroprotection by providing neurotrophic and growth factors, both decreasing the toxicity in the microenvironment and replacing neuronal cells [116]. Consistent with this, it has been shown that stem cell engraftment into SMA mice results in upregulation of the growth factors neurotrophin 3, neurotrophin 4, vascular endothelial growth factor, and nerve growth factor, along with ciliary, brain-derived, and glial-derived neurotrophic factors that improve axonal length of motor neurons [117]. This was accompanied by decreased motor neuron loss, increased muscle mass, and improved motor functions, ameliorating the SMA phenotype and increasing lifespan [116,117,118]. Transplantation of genetically modified induced pluripotent stem cells, obtained from SMA patients’ skin fibroblasts, into SMA mice represents a further step to implement cell therapy in the clinic [117]. However, little is known about the regulatory network and mechanisms that allow stem cells to provide a neuroprotective effect, which remains a significant limitation and hinders the further development of stem cell transplantation as a potential therapy.

### 5.5. Muscle Enhancement

Skeletal muscle wasting and weakness is another prominent feature of SMA. Hence, increasing muscle mass and function is an alternative therapeutic strategy investigated and currently being introduced in clinical trials.

#### 5.5.1. Myostatin Inhibitors

Myostatin is a growth factor, mainly produced by skeletal cells, that negatively regulates muscle growth. Upregulating follistatin expression in preclinical trials, an endogenous inhibitor of myostatin, has shown over 25% increases in muscle mass and lifespan of SMA mouse models [119,120]. However, only modest changes in muscle mass have been reported in mice with severe SMA [121,122]. Inhibition of the myostatin-signaling pathway has also been achieved by blocking the myostatin receptor activin receptor IIB (ActRIIB), improving muscle size and function in SMA mouse models [120,121]. A phase 1a clinical trial is ongoing to investigate BIIB 110, a recombinant ligand of ActRIIB [123]. However, due to the high homology between myostatin and other members of the TGFβ superfamily, especially Activin A, GDF11, and BMPs 9 and 10 [124], as well as other growth factors that also signal through ActRIIB, this non-selective inhibition of myostatin may increase the risk of side effects [125]. Alternatively, targeting the preforms of myostatin and thereby inhibiting the proteolytic cleavage that precedes mature myostatin has proven successful in selectively blocking myostatin activation. SRK-015 (Apitegromab), an anti-myostatin proform monoclonal antibody, has been observed to increase muscle mass and strength in animal models [126], with no safety concerns reported in subsequent clinical trials administering up to 30 mg/Kg apitegromab in healthy participants [127]. Interim results from a phase 2 clinical trial (NCT03921528), known as TOPAZ, currently investigating the safety and efficacy of SRK-015 in patients with 5q SMA type II and type III, were presented at the 2021 World Congress of Neurology. Joint treatment of intravenous injection of 20 mg/Kg apitegromab every 4 weeks together with nusinersen resulted in 60% of the patients achieving a minimum 3-point increase in motor function tests, compared to 27% of patients who received nusinersen alone [128].

#### 5.5.2. Fast Troponin Activators

Fast skeletal troponin activators can improve Ca^2+^ sensitivity of the sarcomere, the contractile unit of muscle, increasing muscle force and function. CK-2127107 (Reldesemtiv) has been shown to improve exercise performance in a rat model of heart failure [129]. Improvements in muscle function have also been reported from phase 1 clinical trials in healthy individuals who received up to 4000 mg/Kg reldesemtiv with only mild side effects unrelated to treatment [130]. A phase 2 clinical trial has also reported increments in skeletal muscle force in patients with 5q SMA types II, III, and IV who received 150 mg/Kg or 450 mg/Kg twice a day [131]. However, only modest changes in two outcome measures from 10 have been reported [131].

### 5.6. Improving Function of Neuromuscular Junction

Neuromuscular junctions (NMJs) are specialized synapses between motor neurons and skeletal muscle fibers. Both structural and functional NMJ abnormalities have been reported in human and animal models of SMA [100], with nearly 50% of patients with 5q SMA types II and III presenting NMJ defects, which seem to be the underlying causes of muscle weakness and increased fatigability [132]. Therefore, enhancing neuromuscular transmission appears to be an alternative therapeutic approach. Pyridostigmine is an FDA-approved acetylcholinesterase inhibitor used in the treatment of myasthenia gravis. Neuromuscular transmission is facilitated by the inhibition of acetylcholine breakdown, resulting in increased neurotransmitter levels at the NMJ [133]. A phase II double-blind, placebo-controlled clinical trial is currently investigating pyridostigmine efficacy in patients with 5q SMA types II, III, and IV [133]. Neuromuscular transmission can also be facilitated by 4-aminopyridine (4-AP), a drug used to treat multiple sclerosis that blocks potassium channels, increasing neurotransmitter release and action potential duration at the NMJ synapse [134]. First identified for its potential therapeutic benefits in SMA in *Caenorhabditis elegans* and *Drosophila melanogaster* screening studies, 4-AP increased muscle size and improved motor function in flies [134] and increased neuromuscular function and rescued the phenotype of *C. elegans* SMA mutant models [135]. However, recent findings have failed to report a clinical benefit in locomotion function after 4-AP treatment in SMA adults [136]. Another amino pyridine, amifampridine phosphate (3, 4 DAP), is being investigated in a phase 2 clinical trial to assess its safety and efficacy in 5q SMA-III patients (NCT03781479). However, results have yet to be reported.

### 5.7. Other Therapeutic Approaches and Combined Strategies

Drug screening and repurposing has revealed drugs acting on different pathways including cytoskeleton dynamics, endocytosis, cell death, mitochondrial, and degradation pathways to increase the availability of SMA. However, most research has been limited to the preclinical stage. Other therapeutic strategies have aimed to upregulate the *SMN2* transcript through the use of histone deacetylase inhibitors, particularly valproic acid, phenylbutyrate, and trichostatin A. Nevertheless, only modest and variable results have been found in the clinic [137,138]. Protein stabilizers to increase SMN protein availability, mainly azithromycin and bortezomib, have shown promising results in animal models of SMA [139,140]; however, clinical trials are also lacking.

Nevertheless, a combination of therapies directly acting to increase SMN protein through splicing or gene replacement mechanisms with other SMN-independent approaches might allow their synergistic action and enhance the overall clinical outcome of the disease. Combined Nusinersen and Zolgensma therapy (both SMN-dependent) in 5q SMA type I patients seems to offer no additional benefits in motor function progression or ventilation over monotherapy with either of these treatments, emphasizing that early treatment is more important than the combined strategy [141]. However, NMJ transmission defects are still reported after 14 months of treatment with nusinersen [142], illustrating the heterogeneity of this disease among patients who could benefit from SMN-independent therapies tackling other mechanisms underlying the disease. Though encouraging results have been observed with regards to muscle mass, motor neuron function, and physical performance in the preclinical stage [139,143], clinical trials have been limited to a small number of participants, therefore leaving the benefits of dual therapy over monotherapy unclear [141,144].

## 6. Challenges and Future Perspectives

The emergence of SMA-specific therapies has not only revolutionized the clinical course of this genetic disease, but it also opens the door to new considerations and challenges. Increased motor neuron preservation and muscle function, followed by attenuation of symptoms and achievement of new motor milestones, indicate a divergence from the established clinical course of disease as milder phenotypes of SMA arise. Therefore, standards of care should be updated accordingly. Nevertheless, implementing updated disease management programmes comes with a significant challenge, since conventional methods are based on decades of observations, yet we are still uncertain about the long-term effects of these novel therapies and how new SMA phenotypes will continue to change in the future.

Small molecules, ASO-based therapy, and genetic therapy have been shown to offer the best clinical benefits when patients are treated before symptoms develop and motor neurons are lost. An early disease onset in 5q SMA types I and 0 illustrates the imperative need for implementing the aforementioned up-to-date strategies that might include newborn or even prenatal SMA genetic screening. Quantitative real time PCR (qPCR) seems to be an effective method for genetically testing newborns [7,145,146]. At present, nine countries have implemented SMA newborn screening (NBS) programs, Taiwan and the US being the leading nations with 90% and 70% of newborns screened for SMA, respectively [4]. Efforts to implement such screening strategies have been largely dominated by Europe [147], where successful implementation has also been achieved in Germany, Belgium, and Italy [4]. Furthermore, approval of national NBS schemes has been granted in the Netherlands and Slovenia, followed by ongoing pilot studies in the UK and Spain [147]. SMA NBS programs are also active in some regions of Australia, Russia, Canada, and Japan [4]. Having a well-established NBS program in place as well as early efforts towards offering genetic screening for SMA in newborns seem to have eased the diligent implementation of SMA NBS in the countries listed above. Taiwan, the only country in the world where NBS is available across the whole nation, began pilot studies in 2014 [9]. Furthermore, including genetic screening for SMA as part of a panel that screens for other conditions such as severe combined immunodeficiency (SCID) might allow for a more feasible implementation of the program, since this multiplexing step could make the process more cost-effective and practical, as suggested by the screening programs of Taiwan, Canada, and Australia [6,9,10]. Nevertheless, ethical concerns and discrepancies between the healthcare systems across countries regarding the selection criteria for patients that should receive priority treatment exist. Immediate treatment is granted for children with homozygous 5q SMA and two *SMN2* copies, whereas those with three *SMN2* copies are categorized as being in a less urgent need for treatment; therefore, they are checked monthly [6,9]. A pilot study in Ontario, however, has developed an algorithm that suggests that individuals with 5q SMA who have four copies of *SMN2* could be selected for treatment based on changes in compound action motor potential (CAMP) measurements [10]. Decrease in CAPM measurements has been proved to precede symptom development of SMA [148]. Therefore, this might be a useful tool in the selection criteria for treatment, providing equality in treatment access to patients for whom the age of disease onset is less certain and who might lose the best clinical benefits of SMA-specific therapies if treated after symptom onset. Finally, an NBS program developed in Australia, where a multidisciplinary team involving clinicians and laboratory staff work closely with psychological and family support on an individual case basis [149], has provided a useful model for establishing integrative screening programs that might offer the best clinical outcomes and quality of life for SMA patients.

Furthermore, dealing with the high costs of these drugs is another factor that will have to be considered not only by patients but also by governments. The first year of treatment with Spinraza was estimated to cost $776,000, followed by nearly half a million dollars for subsequent years [150], and Zolgensma is priced at $2.1 million for a single dose, which represents an economic barrier that can create inequality in access to treatment. Delivering SMA therapy through national healthcare insurance might reduce the gap between patients who can access therapy and those who cannot; however, the elevated therapy costs in addition to those of infrastructure, genetic screening, and logistics programs that have to be in place for effective delivery of treatment should also be faced by the public sector. At present, it has been suggested that these SMA therapies are far from being cost-effective [150]; however, a rather recent introduction of SMA-specific treatments in the market limits our knowledge of whether this will significantly reduce the resource requirements and economic burden that SMA places on the healthcare system in the future. Furthermore, ethical considerations follow for reaching consensus criteria on the patients that should have immediate access to treatment. In spite of *SMN2* copy number directly correlating with disease severity, other factors that can ameliorate symptoms such as genetic modifiers [36,151,152] add to the complexity of identifying those at urgent need of treatment.

Amid the popularity of and strong research focus on already-approved 5q SMA therapies, the potential of other compounds still under development, combinatorial therapies, and SMN-independent clinical strategies might have been overlooked, resulting in discontinuation of the development of some of these [82]. Perhaps, as new milder forms of SMA arise, these alternative strategies might continue to improve the clinical course of SMA, given the multisystemic nature of this disease. Furthermore, the rather recent development of SMA-specific novel therapies has excluded patients with milder forms of SMA, mainly types III and IV, from receiving SMA-specific treatment at an earlier disease onset. These patients, for whom the treatment window for achieving the best clinical outcomes has closed, could also benefit from these alternatives. Moreover, it is important to remember that in spite of recent advances in 5q SMA therapy, we have not found a definitive cure for this disease. At present, research has focused on reversing disease pathology by administering SMA-modifying treatments with the same parameters to a wide range of the SMA population where dose, body weight or ages are irrelevant. However, identifying biomarkers that allow a more accurate diagnosis, prognosis, and assessment of response to treatment could allow for a more tailored delivery of treatment. SMN protein levels, genetic modifiers (including SMN2 copy number, plastin 3, coronin 1c, splicing regulators, and epigenetic differences), electrophysiological changes in muscle function, and muscle quality and composition determined by imaging methods have been suggested as important biomarkers in disease prognosis and response to treatment [153]. Exploring these biomarkers and revealing more as SMA therapy is delivered could ease the process of stratifying patients who would benefit from monotherapy or combined approaches based on their particular disease profile.

SMA therapy will pave the road for the implementation of next-generation therapies to treat other genetic diseases in the future. It is therefore urgent that, despite the uncertainty and challenging factors surrounding the subject, both the political and healthcare sectors work together on the legal, social, economic, and logistic foundations that will enable the nation-wide implementation of SMA treatment.

## 7. Conclusions

In the last decade, the development of innovative *SMN* replacement and/or splice modulation strategies has provided therapeutic options for children diagnosed with 5q SMA, despite its extremely high cost. These approved therapies extend life expectancy and improve neuromuscular functions. However, regardless of the pioneering nature of these therapeutic strategies, it is becoming obvious with hindsight and long-term follow-up of the first treated patients that SMN protein replacement is not a cure, paving the way for development of the next generation of therapies, including *SMN*-independent therapies and combinational therapies. After a few years of clinical experience, we have found that the timing of treatment is critical. The earlier the disease is detected, the better the response to *SMN* replacement therapy. For this reason, the gold standard for SMA treatment should involve neonatal genetic screening, as currently practiced in limited countries in the world. The hope is that neonatal genetic screening will improve early diagnosis and therapeutic strategies for SMA patients.

## Figures and Tables

**Table 1:** Nine SMA newborn screening programs worldwide showing the number of SMA patients, the total of neonates tested, and the incidence rates.

Country	SMA Patients	Neonates Tested	Incidence Rate	Reference
Italy	12	58,558	1:4880	[4]
Germany	43	297,163	1:6911	[5]
Australia	19	202,388	1:10,652	[6]
The United States	180	2,395,718	1:13,310	[7]
Belgium	9	127,329	1:14,148	[8]
Taiwan	20	419,102	1:20,955	[9]
Canada	5	139,810	1:27,962	[10]
Russia	0	12,000	0	[4]
Japan	0	22,209	0	[11]
Worldwide	288	3,674,277	1:12,758	[4]

## Data Availability

Not applicable.
